# Stuttering and Word-Finding Difficulties in a Patient With COVID-19 Presenting to the Emergency Department

**DOI:** 10.7759/cureus.11774

**Published:** 2020-11-29

**Authors:** Nathan Morrison, Joshua Levy, Talia Shoshany, Aaron Dickinson, Michael Whalen

**Affiliations:** 1 Internal Medicine, Philadelphia College of Osteopathic Medicine, Philadelphia, USA; 2 Internal Medicine, Lankenau Medical Center, Wynnewood, USA; 3 Emergency Medicine, Lankenau Medical Center, Wynnewood, USA

**Keywords:** covid 19, sars-cov-2, stuttering, word finding impairments, neurological signs and symptoms, keywords: magnetic resonance imaging, case report, emergency department, coronavirus

## Abstract

Coronavirus disease 2019 (COVID-19) was designated as a global pandemic by the World Health Organization (WHO) on March 11, 2020. The Cochrane Database of Systematic Reviews documents that COVID-19 has a wide range of common symptoms, which have made it difficult to characterize the disease. To date, the neurological symptoms of stuttering and word-finding difficulties have not been reported in confirmed COVID-19 cases. This case report describes the clinical course of a 53-year-old female that presented to the emergency department (ED) twice with varying symptoms consistent with COVID-19. At the second ED visit, she complained of new-onset stuttering and word-finding difficulties and tested positive for COVID-19 using the polymerase chain reaction (PCR) nasopharynx test. When contacted, the patient stated that her speech issues persisted at least seven days after discharge from her second ED visit. As a result, the virus may cause symptoms of an acute neurological event and should be taken into diagnostic consideration. These neurological findings may be explained by the recent discovery of the COVID-19 spike protein’s ability to destabilize the blood-brain barrier (BBB) and enter the central nervous system (CNS). Increased classification of unrecognized COVID-19 symptoms and complications may aid in the characterization, surveillance, and prevention of the disease.

## Introduction

Severe acute respiratory syndrome coronavirus 2 (SARS-CoV-2) has become an increasingly ordinary consideration for the practice of medicine. As of November 2020, there are 46,379,835 confirmed global cases, 1,198,688 confirmed deaths, and a case fatality rate of approximately 2.59% [1]. The range of associated symptoms for COVID-19 has been well-documented by the Cochrane Database of Systematic Reviews. Although there are currently no pathognomonic signs or symptoms of COVID-19, the database reported that common symptoms include (percentage of confirmed cases): cough (50%), subjective or clinical fever (43%), myalgia (36%), headache (34%), dyspnea (34%), pharyngitis (20%), diarrhea (19%), nausea and vomiting (12%), anosmia or ageusia (<10%), abdominal pain (<10%), and rhinorrhea (<10%) [2-3]. This case report describes a patient who presented to the emergency department (ED) on two separate occasions in a four-day time period. During her first ED visit, she complained of generalized weakness, fevers, and a productive cough but had a negative qualitative SARS-CoV-2 nucleic-acid amplification test (NAAT). At her second ED visit, her chief complaint was stuttering and word-finding difficulties. At this visit, she received a positive SARS-CoV-2 polymerase chain reaction (PCR) nasopharynx test.

## Case presentation

A 53-year-old female with a past medical history of hypertension and migraines presented to the ED twice over a four-day time period. During her first ED visit, she was brought in by her sister for concern of generalized weakness, chills, a fever of 38.2°C (100.8°F), productive cough, nausea, and diarrhea for six days. The patient’s vital signs on presentation to the ED revealed a blood pressure of 116/82 mmHg, pulse of 120 beats per minute (bpm), respiratory rate of 16 breaths per minute (br/min), oxygen saturation of 98% on room air, and temperature of 38.2°C (100.8°F). Her laboratory testing revealed normal troponin, lipase, and magnesium levels. Her basic metabolic panel, hepatic function panel, complete blood count with differential, and electrocardiogram (ECG) were unremarkable. Her chest X-ray displayed a left lower lung zone airspace opacity, likely representing developing pneumonia. After evaluation, her clinical presentation was suggestive of a viral left lower lobe pneumonia. She was prescribed a three-day course of 500 milligrams of azithromycin and discharged home. Her qualitative SARS-CoV-2 NAAT, influenza A/B, and respiratory syncytial virus (RSV) PCR were negative at that time. 

**Figure 1:**
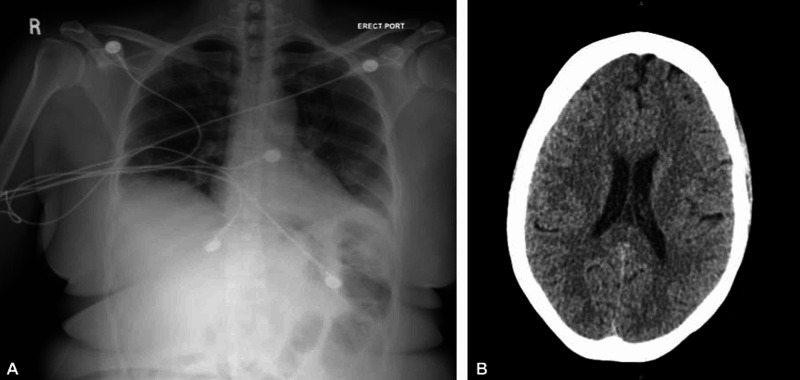
A. Anteroposterior view chest X-ray at the patient’s second ED visit; B. CT of the head without contrast at the patient’s second ED visit A. This image does not reveal any typical active COVID-19 radiologic signs. There was a limited inspiratory effort, no pulmonary vascular congestion or edema, no pulmonic infiltration, no atelectasis or pleural effusions demonstrated. The cardiomediastinal silhouette is normal in size and configuration, with the bony thorax intact. B. There are no signs of acute intracranial hemorrhage, acute infarct in a major vascular territory, or mass-effect. ED: emergency department; CT: computed tomography

**Figure 2:**
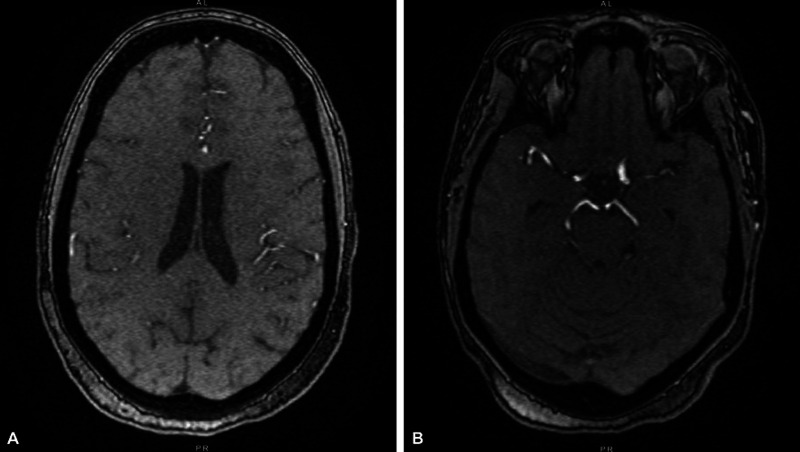
A. T1-weighted MRI of the brain without contrast at the patient’s second ED visit; B. MRA of the brain without contrast at the patient’s second ED visit A. This unenhanced MRI image of the brain is within normal limits. B. The circle of Willis had no evidence of major vessel stenosis, cutoff, narrowing, or aneurysmal dilatation. The vertebral arteries appear co-dominant. ED: emergency department; MRI: magnetic resonance imaging; MRA: magnetic resonance angiography

Four days later, the patient presented to the ED for a second visit. Her initial symptoms had mostly improved, although she had concerns for newly developed stuttering and word-finding difficulties. She had no issues with her speech in the past and was last witnessed at her baseline speech four days prior by her sister. The patient’s vital signs on presentation at the second ED visit revealed a blood pressure of 133/66 mm Hg, pulse of 100 bpm, respiratory rate of 16 br/min, oxygen saturation of 97% on room air, and temperature of 36.6°C (97.9°F). On physical examination, the only notable findings were generalized weakness and stuttering. Other notable testing revealed a normal ECG, troponin, thyroid-stimulating hormone (TSH), folate, vitamin B12, lipid panel, and glycated hemoglobin (HBA1c) of 5.0%. Imaging via an anteroposterior chest X-ray showed no active disease (Figure 1A). Further imaging with a non-contrast computed tomography (CT) of the head revealed no masses or intracranial hemorrhages (Figure 1B). She tested positive for COVID-19 on admission protocol via a SARS-CoV-2 PCR nasopharynx test. The patient was not started on Remdesivir or steroids at that time for treatment due to mild COVID-19 signs. She was monitored with pulse oximetry and maintained adequate oxygen saturation on room air throughout her two-day admission. Neurology was consulted by the inpatient medicine team for concern of a stroke, but magnetic resonance imaging/angiography (MRI/MRA) of the head and neck were negative for acute pathology (Figure 2A, Figure 2B). The anomic aphasia (word-finding difficulties) and stuttering were due to an unclear etiology but plausibly a response to the COVID-19 infection according to neurology. The patient was contacted on the second and seventh days following discharge and stated that she only had mild improvement in stuttering and word-finding.

## Discussion

Several neurological signs, symptoms, and complications of confirmed COVID-19 cases that have been reported include the following: headache, dizziness, myalgia, anosmia, encephalopathy, encephalitis, necrotizing hemorrhagic encephalopathy, stroke, epileptic seizures, and Guillain-Barre syndrome [4]. No known cases have reported stuttering and word-finding difficulties. Although this patient’s first SARS-CoV-2 NAAT was negative four days prior to her second ED visit, it is possible that it could have been a false-negative test. The false-negative rate has been reported up to 40% in SARS-CoV-2 NAATs that are performed within 20 days of symptom onset. It is also recommended that if clinical suspicion of COVID-19 remains following a negative test, a repeat test should be performed [5-6]. Considering this patient had varying symptoms consistent with COVID-19 over a 10-day period, her contraction of COVID-19 is proposed to be approximately when her initial symptoms began.

Aphasia is caused by damage to a portion of the brain that is located near Broca’s or Wenicke’s areas. Aphasia usually occurs in an acute setting, following a stroke or head injury. Slowly progressing aphasia is more commonly associated with brain tumors or neurological disease. CT and MRI are often used to diagnose aphasia and determine the location of brain injury [7]. In this case report, acute stroke was ruled out by brain CT, MRI, and MRA. Although the patient had a history of migraines, complicated migraine was a less likely etiology of the stuttering since the patient did not present with a significant headache associated with the change in speech. This case report suggests that the patient’s anomic aphasia and stuttering were due to a response to the COVID-19 infection.

Stuttering is caused by an impaired ability of the basal ganglia in the brain to produce timing cues of motor speech [8]. A recent study by Buzhdygan et al. found that the SARS-CoV-2 spike protein can destabilize the blood-brain barrier (BBB) and trigger a proinflammatory state at the brain endothelium [9]. This discovery could help explain the coronavirus’s ability to cross the BBB, enter the central nervous system, and produce a response at the brain endothelium. Notably, it has been determined that viruses are capable of creating cross-reactive epitopes with host self-proteins [10]. It is also important to consider that most human coronaviruses (OC-43, 229E, MERS, and SARS) have the potential for neuroinvasion and can cause neurological symptoms [4]. The spike protein could play a role in the ability of COVID-19 to interact with the basal ganglia and produce stuttering and anomic aphasia in infected patients.

## Conclusions

COVID-19 has been associated with non-specific symptoms such as cough, subjective or clinical fever, myalgia, headache, dyspnea, sore throat, diarrhea, nausea and vomiting, loss of taste or smell, abdominal pain, and rhinorrhea. In cases of patients that present with unexplained acute neurological symptoms, such as stuttering or word-finding difficulties, negative imaging studies, and no other complaints, it may be important to rule out COVID-19 prior to disposition in acute care settings. If overlooked, these patients may be misdiagnosed due to a lack of well-known symptoms of COVID-19. Early identification in these patients can help initiate patient quarantine, prevent contact-spread, and begin early treatment. Further investigation and case reports may highlight whether the stuttering and word-finding difficulties are an early, late, or persistent manifestation of COVID-19 infections.
